# Cyanobacterial Light-Driven Proton Pump, *Gloeobacter* Rhodopsin: Complementarity between Rhodopsin-Based Energy Production and Photosynthesis

**DOI:** 10.1371/journal.pone.0110643

**Published:** 2014-10-27

**Authors:** Ah Reum Choi, Lichi Shi, Leonid S. Brown, Kwang-Hwan Jung

**Affiliations:** 1 Department of Life Science and Institute of Biological Interfaces, Sogang University, Seoul, Korea; 2 Department of Physics, University of Guelph, Ontario, Canada; University of Freiburg, Germany

## Abstract

A homologue of type I rhodopsin was found in the unicellular *Gloeobacter violaceus* PCC7421, which is believed to be primitive because of the lack of thylakoids and peculiar morphology of phycobilisomes. The *Gloeobacter* rhodopsin (GR) gene encodes a polypeptide of 298 amino acids. This gene is localized alone in the genome unlike cyanobacterium *Anabaena* opsin, which is clustered together with 14 kDa transducer gene. Amino acid sequence comparison of GR with other type I rhodopsin shows several conserved residues important for retinal binding and H^+^ pumping. In this study, the gene was expressed in *Escherichia coli* and bound all-*trans* retinal to form a pigment (λmax  = 544 nm at pH 7). The pK_a_ of proton acceptor (Asp121) for the Schiff base, is approximately 5.9, so GR can translocate H^+^ under physiological conditions (pH 7.4). In order to prove the functional activity in the cell, pumping activity was measured in the sphaeroplast membranes of *E. coli* and one of *Gloeobacter* whole cell. The efficient proton pumping and rapid photocycle of GR strongly suggests that *Gloeobacter* rhodopsin functions as a proton pumping in its natural environment, probably compensating the shortage of energy generated by chlorophyll-based photosynthesis without thylakoids.

## Introduction

Archaeal rhodopsins are light-responsive seven transmembrane proteins that bind all-*trans*-retinal as chromophore. They are type I rhodopsins, in contrast to 11-*cis*-retinal-based type II rhodopsins, such as the animal visual pigments [1], [2]. Bacteriorhodopsin (BR) and halorhodopsin (HR) are light-driven proton and chloride pumps. They enable *Halobacterium salinarum* to grow phototrophically by establishing electrochemical ion gradients across the cell membrane to provide cellular energy and pH balance in the extreme ionic environmental conditions. Sensory rhodopsins (SRs) I and II, however, act as attractants for orange (SRI) and repellent photoreceptors for UV-blue (SRI) and green light (SRII), respectively. The covalent attachment of the chromophore (via a protonated Schiff base to a lysine residue in the helix G) and interactions with selected amino acids within the binding pocket allow a tuning of the absorption wavelength over a wide spectral range. Light-induced *trans-cis* isomerization of the chromophore triggers a photocycle that includes Schiff base deprotonation (in BR and SRs) and protein conformational changes, the former evident from a strong photochromic shift of this intermediate species [1].

In recent years, evidence has emerged that the occurrence of type I rhodopsins has spread beyond the borders of the *Archaea* domain [3]–[5] Type I archaeal rhodopsins are found in phylogenetically diverse microorganisms, including halo-archaea, proteobacteria, cyanobacteria, fungi and algae [1], [6]–[10]. Among those, cyanobacteria are known to use light as a source of energy. For example, *Anabaena* rhodopsin is photosensory receptor that is spectrally tuned to relay information to the cell regarding the intensity and color of light in the environment, possibly to adjust a composition of the light-harvesting machinery [9], [11], [12].

While the exact origin of oxygenic photosynthetic organisms is not known, we are aware of continuity/similarity of reaction center complexes in photosynthetic bacteria [13]. Also, we do not know the origin of type I rhodopsins, which represent a very simple energy production system and various sensory receptors for the light-dependent signal relay. Since the discovery of a type I rhodopsin in the early seventies, only recently the co-existence of a proton transporting rhodopsin and photosynthetic pigments has been reviewed in [3], [5]. One can try to understand the emergence of two kinds of energy production system in one organism, either through lateral gene transfer or the last universal common ancestor. The appearance of oxygenic photosynthetic organisms determined the direction of global biological evolution through an increase in the oxygen concentration of the Earth atmosphere and developing a more efficient energy production through photosynthetic machinery. There will be many challenges to understanding the discontinuity of the presence of rhodopsins using the photosynthetic microbes in which both systems coexist. One possible approach is to search an organism that has retained primitive properties, such as photosynthetic protein complexes, their localization, reaction processes, and so on. It was the adherence to this approach that led us to focus on the cyanobacterium *Gloeobacter violaceus*.

*G. violaceus* is a rod-shaped unicellular cyanobacterium isolated from calcareous rock in Switzerland [14]. According to phylogenetic analysis based on the 16S rRNA sequence, it diverges very early from the common cyanobacterial phylogenetic branch, suggesting that it may retain some of the primitive properties of early cyanobacteria [15]–[17]. However, it has several differences from typical cyanobacteria. They lack thylakoid membrane development [14], [18], having its photosynthetic and respiratory systems located in the cell membranes instead of thylakoid membranes, where the machinery is found in other cyanobacteria [14]. This means that components facing the lumen in the cytoplasm in other cyanobacteria are exposed to periplasm in *Gloeobacter*, thus the photosynthetic electron transfer system should co-exist in the cytoplasmic membrane with a respiratory system by sharing some components [19]. The morphology of phycobilisomes is distinct from other cyanobacteria as well. Phycobiliproteins form rod-shaped elements and these elements form bundle-shaped aggregates, which attach to the cell membranes from the cytoplasmic side [16], and oxygen evolution is thus mediated in the periplasmic space.

A gene encoding new rhodopsin with high sequence homology to the previously described type I rhodopsins of *Archaea* and *Eucarya* was found in this cyanobacterium through genome sequencing [19]. In this study, we identified, heterologously expressed, and functionally characterized the gene product that acts as a light-driven proton pump in *G. violaceus* PCC7421. The pigment has been named GR (Gloeorhodopsin). While this manuscript was in preparation, two FTIR studies [20], [21] were published, reporting on other spectroscopic features consistent with the proton-pumping function of GR, in full agreement with the present study. In addition, here we report that the rhodopsin is heterologously expressed and test the function in the native host.

## Experimental Procedures

### Source of strains and culture

*Gloeobacter violaceus* PCC 7421 was obtained from the Culture Collection of Algae and was grown in a Z-medium under photoautotrophic conditions at 25°C. The light source was a fluorescent lamp (FL20SD) at an intensity of 10 µmol m^−2^s^−1^.

### Cloning of *Gloeorhodopsin* gene and site-directed mutagenesis

In order to clone the Gloeorhodopsin gene, we used Trizol to lyse the cells and purify a genomic DNA. The region to be analyzed was amplified using standard PCR approaches. PCR was performed using the Gloeo-for primer (5′ATGTTGATGACCGTATTTTCTTCTGC3′) and Gloeo-rev primer (5′CTAGGAGATAAGACTGCCTCCCCG3′). Double-stranded amplification product (30 amplification cycles of 95°C (1 min), 53°C (1 min), 72°C (2 min 30 sec)) was purified by isolating the desired band after 1% agarose gel electrophoresis in 1X TAE buffer. The pKJ900 plasmid encoding Gloeoopsin was used as a template for site-directed mutagenesis. Site-directed mutagenesis was carried out with the two-step megaprimer PCR method with *Pfu* polymerase [22]. To prove the function of GR, we designed three site-directed mutants: D121N, E132Q, and E132D. These mutations were aimed at modifying the translocation of protons in this microbial rhodopsin.

### Bacterial strains and plasmids

*Escherichia coli* strain DH5α was used for cloning the WT/mutant Gloeorhodopsin genes and transformants were grown in LB (Luria-Bertani) medium in the presence of ampicillin (50 µg/mL) (USB Corp.) at 35°C. *E. coli* strain β/UT was constructed by transforming plasmid pORANGE [23] into *E. coli* UT5600, resulting in ability to synthesize ß-carotene. The plasmid pKJ900 carrying the *Nco*I/*Pme*I (ß-*diox*) [23] and *Nde*I/*Not*I (GR) fragment was used.

### Expression and purification

Gloeorhodopsins were expressed under the lacUV5 promoter and ß-*diox* was expressed under pBAD promoter in *E. coli* strain UT5600 or β/UT. UT5600 were induced with 1 mM IPTG for 6 h at 35°C and all-*trans* retinal was added to final concentration of 5∼10 µM. β/UT cells were induced with 1 mM IPTG and 0.2% (+)-L-arabinose for 24 h at 35°C. The membranes were solubilized with 1% n-Dodecyl-β-D-Maltopyranoside(DDM) and purified with Ni^2+^-NTA agarose (Qiagen). The purified WT and mutant GR samples contained 0.02% DDM [24].

### Absorption spectroscopy and pK_a_ determination

UV/VIS spectroscopy was used to measure the absorption spectra of the purified GR in DDM. To make the samples suitable for absorption measurements, a treatment was applied with DDM [11]. Briefly, the membrane pellets were treated with 0.5% (w/v) DDM for 3 min and centrifuged at 4000×g for 0034 min, yielding a pellet of large membrane aggregates, which were discarded. The supernatant was centrifuged at higher speed (20,000xg for 20 min), producing a pellet of membranes containing GR, which upon re-suspension gave a clear sample suitable for absorption measurement. The membranes were incorporated into poly-acrylamide gels, which were washed in de-ionized water (overnight or longer) to remove the residual detergent and polymerization agents, and then soaked in an appropriate buffer (>2hrs) [25]. The absorption spectra of the wild-type and mutants in DDM were recorded at various pH with Shimadzu UV visible spectrophotometer (UV-2450). For spectroscopic titration, the GR samples in the poly-acrylamide gel were first soaked in 3-mix buffer (pH ca. 12). Then the pH was adjusted to the desired value by the addition of a very small amount of HCl, followed by the measurement of absorption spectrum. The data were fitted with functions containing pK_a_ components (y = A/(1+10^pH-pKa^)). The photocycle kinetics were measured using flash-photolysis apparatus, using 532 nm Nd-YAG laser (Minilite, Continuum, 7 ns flash) for excitation.

### Proton pumping measurements

Sphaeroplast vesicles of *E. coli* expressed GR WT and mutants were collected at 30,778×g for 1 hr at 4°C (Beckman XL-90 ultracentrifuge) and washed with 10 ml of solution I (10 mM NaCl, 10 mM MgSO_4_·7H_2_O, 100 mM CaCl_2_) and resuspended in 3 ml of solution II [26]. Samples were illuminated through the short wave cutoff filter (Sigma Koki SCF-50S-44Y) and sphaeroplasts of *Gloeobacter* whole cells were illuminated through various filters (Sigma Koki SCF-400F40-10, 450F40-10, 500F40-10, 550F40-10, 600F40-10, 650F40-10, 700F40-10) and various light intensity (transmitted filter 100%, 76%, 30%). The pH changes were monitored using Horiba pH meter F-51. The pH data were transferred and recorded automatically with Horiba Navi program.

### GR specific antibody preparation and immunoblot assay

*E. coli* cell lysates containing 40 µg of protein were prepared. The quantity of proteins was determined with Bio-Rad D_c_ protein assay kit. Anti 6xHis polyclonal mouse antibody at a 1∶6,000 dilution was used as the primary antibody, and HRP conjugated goat anti-mouse IgG antibody (SantaCruz) at a 1∶15,000 dilution was used as the secondary antibody. Reactive bands were visualized with Western Lightning Chemiluminescence Reagent Plus (PerkinElmer Life Science).

In order to produce the primary antibody, *Gloeobacter* rhodopsin was expressed in *E. coli* and purified with Ni^2+^-NTA resin. Purified GR was further separated by SDS-PAGE. Then, the protein bands were chopped with a razor. The chopped proteins in poly-acrylamide block were ground with plastic homogenizer. These protein mixtures were injected twice under the skin of mice. The anti-*Gloeobacter* rhodopsin antibody from mice was used at 1∶100 dilution as the primary antibody. The HRP conjugated goat anti-mouse IgG antibody at a 1∶10,000 dilution was used as the secondary antibody.

### ATP assay

Cultured WT *G. violaceus* cells and the photosynthesis inhibitor, DCMU ((3-(3,4-dichlorophenyl)-1,1-dimethylurea, 5×10^−5^mol l^−1^)-treated *G. violaceus* cells were used for this assay. Each cells were illuminated>450 nm (Sigma Koki filter) for 5 min. The measurement of the ATP concentration of the sonicated cells followed the vendor's protocol that is ATP assay kit (ATP Bioluminescent Assay Kit) obtained from Sigma Aldrich Inc.

All samples were measured in a MicroLumat Plus LB 96V luminometer (Berthold Technologies, Promega Readit Technology). 6.25×10^−17^ ∼ 1×10^−15^ M ATP solutions were used for obtaining the standard curve.

## Results

### Phylogeny

**Figure 1:**
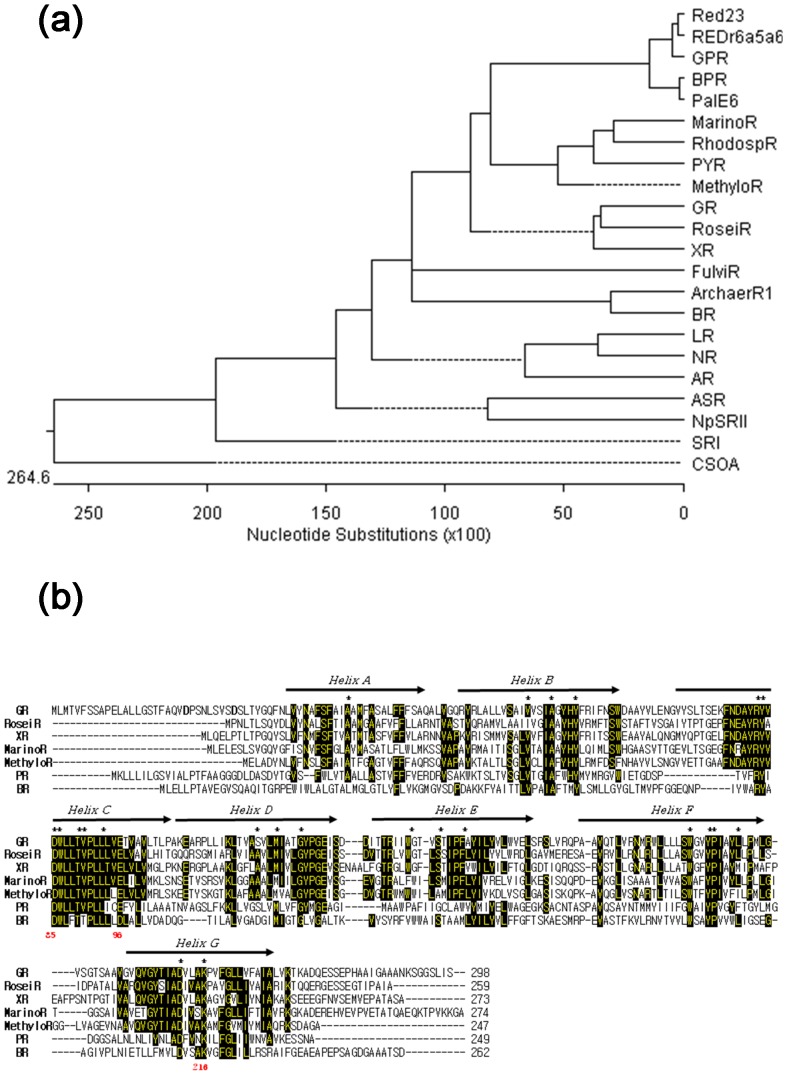
Pylogenetic tree and alignment. (a) Phylogenetic tree of 22 microbial opsin sequences, representing phylogenetic relationship between *Gloeobacter* rhodopsin and related proteins from archaea to algae. Analysis was conducted by ClustalW. A dotted line on a phenogram indicates a negative branch length, a common result of averaging by the DNA STAR software. GR: *Gloeobacter* rhodopsin (accession number; NP_923144). Red23 and REDr6a5a2 were collected from Red Sea. GRP/BPR: green/blue-absorbing PRs. PalE6 was collected from Antarctic ocean. RhodospR: rhodopsin from *Rhodospirillales sp*. XR: Xanthorhodopsin from *Salinibacter ruber*. FulviR: rhodopsin from *Fulvimarina*. ArchaerR1: Archaerhodopsin 1. PYR: rhodopsin from *Pyrocystis lunula*. BR: *Halobacterium salinarum* bacteriorhodopsin. RoseiR: rhodopsin from *Roseiflexus sp*.MarinoR: rhodopsin from *Marinobacter*. MethyloR: rhodopsin from *Methylophilales*.LR: rhodopsin from *Leptosphaeria maculans*. NR: rhodopsin from *Neurospora crassa*. AR: rhodopsin from *Acetabularia acetabulum*. SRI: *H. salinarum* sensory rhodopsin I. NpSRII: *Natronomonas pharaonis* sensory rhodopsin II. ASR: sensory rhodopsin from *Anabaena (Nostoc) sp.*CSOA: *Chlamydomonas reinhardtii* sensory rhodopsin A. (b) Alignment of the sequences of GR, XR, PR, RoseiR, MarinoR, MethyloR and BR. GR contains the functionally important residues for proton transport, including homologues of Arg82, Asp85, Trp86, Asp96, Trp182, Tyr185, Asp212, and Lys216, with some of the numbering and helical segments (marked by arrows) for BR. There are 22 residues (marked with black boxes) common to all seven proteins (in one case, Asp/Glu substitutions). The Asp121 (Asp85 in BR) should be the proton acceptor of the retinal Schiff base. The Glu132 (Asp96 in BR) may be the proton donor.

A phylogenetic comparison with archaeal rhodopsins placed Gloeorhodopsin(GR) on an independent branch, with moderate statistical support for an affiliation with ion pumping rhodopsins (Fig. 1A). Notably, GR is close to Xanthorhodopsin (XR) that has antenna pigment for proton pumping in *Salinibacterruber* [27], and a rhodopsin from *Roseiflexussp*. that a filamentous anoxygenic phototrophic bacteria [28]. From the alignment of the sequences of XR, BR, PR, RoseiR, MarinoR, MethyloR, PYR and GR, the retinal must be localized in the path of proton translocation in GR, too (Fig. 1B). According to result of multiple sequences alignment, the residue Asp121 (Asp85 in BR) can be the proton acceptor of the retinal Schiff base during light-driven proton transport by GR. The major member of the proton-release group, Arg118 (Arg82 in BR) is conserved, and the Glu132 (Asp96 in BR) may be the cytoplasmic proton donor. 137 residues (50%) are identical in GR and XR [27], [29], where the carotenoid in the dual light-harvesting system increases the cross section for light absorption and extends it to the spectral region, in which the absorption of the retinal chromophore is low [27]. Surprisingly, *Gloeobacter* rhodopsin binds salinixanthin, which was extracted from *Salinibacter ruber* [30] and echinenone from *G. violaceus* that both are contains 4-keto group [49]. So, it is possible that GR also has a light-harvesting carotenoid antenna like XR [27], [29]–[31].

### Absorption spectroscopy and pH titrations

**Figure 2:**
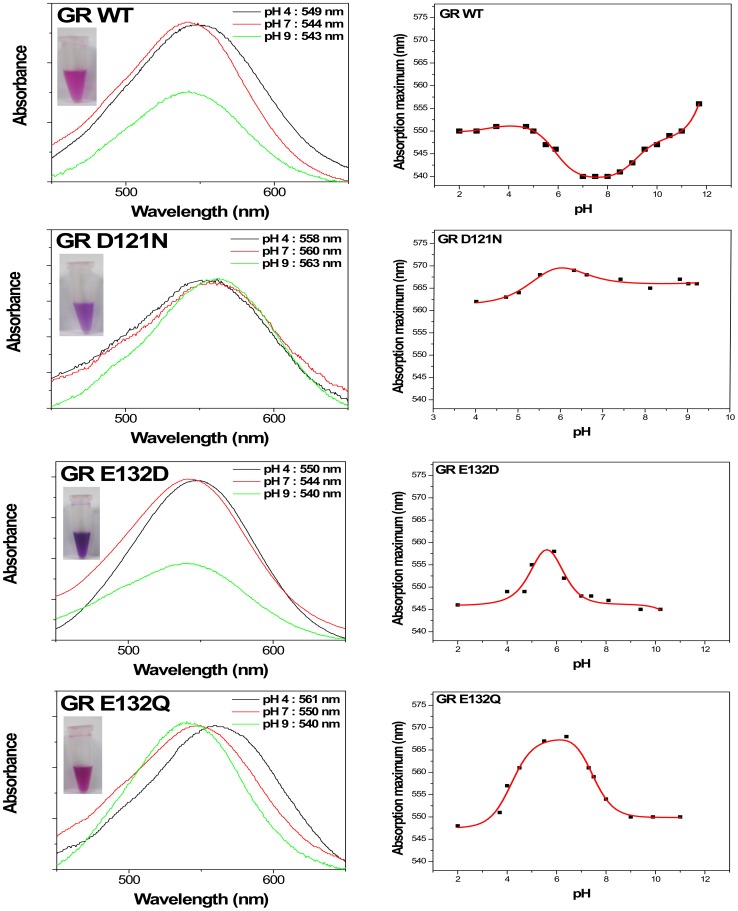
Absorption spectra and titration curves of GR and its mutants. Insets show photos of purified WT and mutant GR in 50 mM Tris (pH 7), 150 mM NaCl, and 0.02% DDM. The pH titration curves indicate the pH dependence of the absorption maxima of broad spectral range. The pK_a_ values are shown in the table 1.

The purified mutants of diverse colors (Fig. 2 inset) were isolated for the biophysical measurements. Absorption maxima of each purified pigment were measured. Microbial rhodopsins in general exhibit two spectral forms in a proton dependent equilibrium [32], [33], so absorption maxima of each pigment were measured in acidic state (pH 4, proton acceptors in the protonated form) and alkaline state (pH 10, proton acceptor is in the deprotonated form). The absorption maximum of the WT GR at pH 7 is 544 nm (Fig. 2). Its 100 nm half-bandwidth is typical of retinylidene protein absorption spectra found in other rhodopsins. The alkaline species, in which the primary retinylidene Schiff base counterion (Asp121 in GR) is unprotonated, have λmax at 543 nm, and the red-shifted acidic species, in which this residue is protonated, have λ_max_ at 549 nm. Comparing the mutant D121N and E132Q with the wild-type GR at pH 7, we observed the absorption maximum of the WT at 544 nm shifted to 560 nm (D121N) or 550 nm (E132Q). An interesting difference in the shift of the absorption bands of homologous mutants is seen in BR and PR. Unlike in BR and PR, not the absence of the red shift of the absorption band but a substantial red shift is seen in GR E132Q, the cytoplasmic proton donor mutant. Such red shift could originate either from the change in the spectrum of the alkaline form or from the change in the pK_a_ of acid-to-alkaline transition (or both). The absorption maximum of another E132 mutant, E132D, is WT-like, at 544 nm at pH 7 (Fig. 2).

**Table 1:** Absorption maximum and pK_a_ value of wild-type and various GR mutants.

Opsin type	GR WT	GR D121N	GR E132D	GR E132Q
**λ_max_ (nm)**	pH 4: 549	pH 4: 558	pH 4: 550	pH 4: 561
	pH 7: 544	pH 7: 560	pH 7: 544	pH 7: 550
	pH 9: 543	pH 9: 563	pH 9: 540	pH 9: 540
**pK_a_**	5.9	6.0	5.4	4.2
	9.2	11.0	6.1	7.2

The absorption maxima of the wild type, D121N, E132Q, and E132D are shown in Fig. 2 as a function of pH. The spectral shift observed at various pH ranges showed much smaller than other microbial rhodopsins, but it could be fitted to yield pK_a_ 5.9. This pK_a_ would be attributed to the primary proton acceptor and a part of the Schiff base counterion, Asp121. Interestingly, there is a progressively fluctuation within the entire tested range from pH 2 to 12 unlike other microbial rhodopsin. There are obviously opposite fluctuations compare to WT in the mutants, D121N, E132Q, and E132D, a part of the Schiff base counterions, indicating their positions close from Schiff base and/or a stronger interaction of these residues with the Schiff base. Among the mutants, each absorption maxima of nonionizable D121N or E132Q is shifted to longer wavelengths than WT. The reason for the fluctuations in the various pH for the mutant proteins is not completely understand until now. When the Asp-85 in BR homolog was replaced with a noninoizable residue in the GR that is D121N mutant, there is no big major transition and pH dependence of the absorption maximum changes, indicating that there is a very small protonation/deprotonation of unknown charged residues.

### Proton translocation in heterologous vesicle

**Figure 3:**
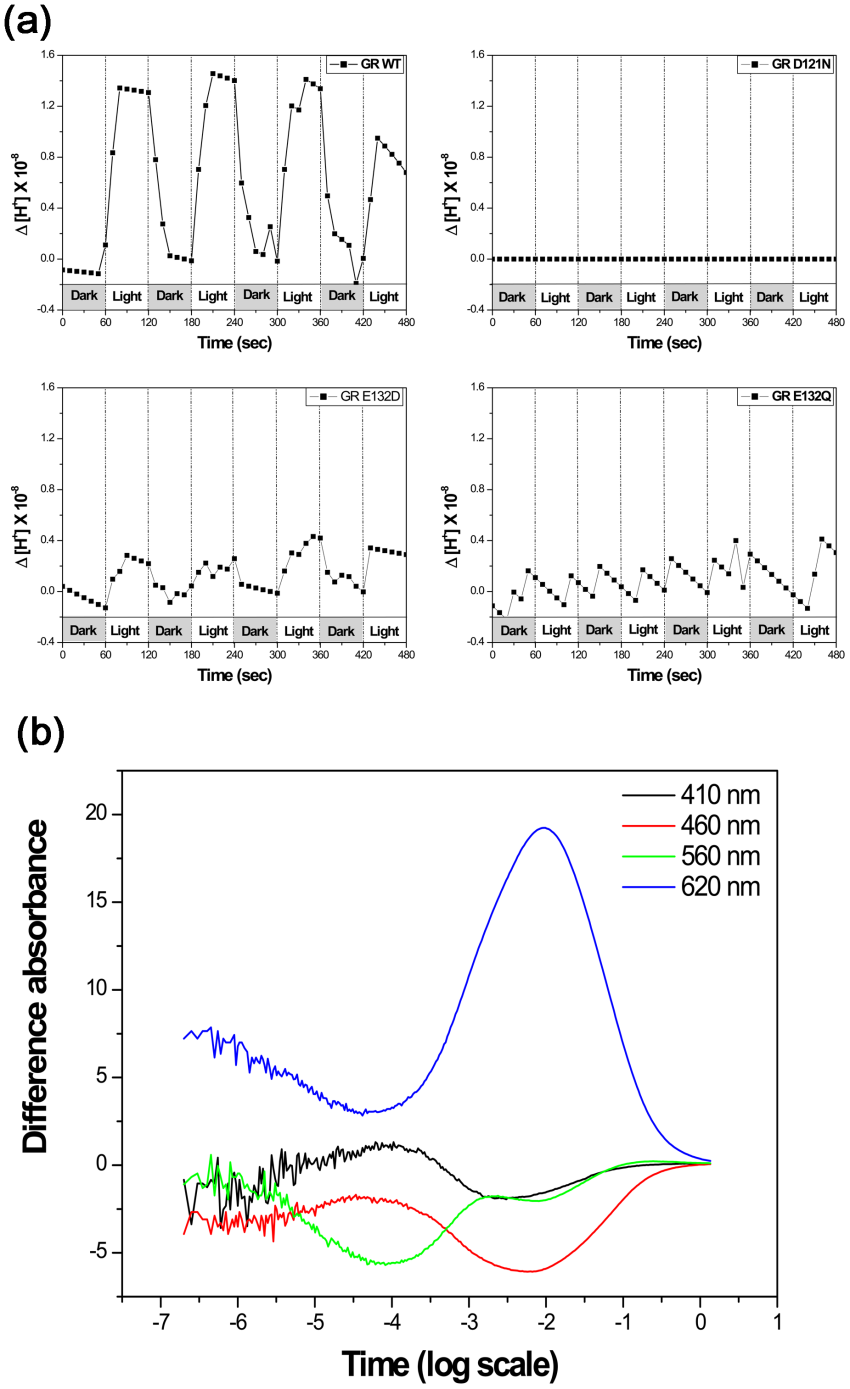
Proton pumping activity and photocycle kinetics. (a) Light induced proton fluxes in *E. coli* vesicles containing wild type and mutant GR. Initial pH values were adjusted to7. The buffering capacity was calibrated and the pH changes were recalculated into proton concentrations. (b) Photocycle kinetics of the wild-type GR measured in polyacrylamide gel-encased, DM-treated membranes at 22°C, in 100 mM NaCl, 50 mM KH_2_PO_4_, pH 8. This figure gives overallcharacteristics of the photocycle with the differential absorption measured at 620 nm for theK and O intermediates, 410 nm for the M intermediate, 460 nm for the L intermediate, and 560 nm for the parent state and the N intermediate. See the full photocycle description in [20].

The wild-type GR in *E. coli* membranes is capable of proton pumping under the light (Fig. 3A) like PR [34]–[37]. While the proton transport capability of the E132D mutant was detectable, but was lower than that of the wild-type, possibly due to the slower photocycle turnover [20]. In accord with the prediction from homology that during the initial stages of the GR's photocycle a proton is transferred from the Schiff base to the deprotonated carboxyl group of Asp121, the replacement of Asp121 by Asn completely abolished proton transport activity. The E132Q mutant was intensively studied, as Glu-132 has an essential role in the proton pumping mechanism of the GR, being the proton donor for the Schiff base. The E132Q mutant shows no proton pumping in the light-activated state, which may be ascribed to the dramatic slow down of its photocycle as well as high 13-*cis*-retinal content of the dark state [20], [21].

### Overall character of the photocycle of GR

Similar to other ion transporting rhodopsins, such as BR [38], [39] and PR [26], the photocycle turnover of GR is rather fast. The wild-type GR showed a faster photocycling rate than sensory rhodopsin (see Fig. 3B with the data at pH 8, the rate is even faster at lower pH, not shown). Rapid photo-cycling rate is typical for the ion transporting rhodopsins vs slow rate (t_1/2_>150 msec) in sensory rhodopsins [40], [41]. The overall characteristics of the photocycle of GR are similar to those of PR and XR. Specifically, *Gloeobacter* rhodopsin has a very low amplitude of the M and a very high concentration of the O intermediate in the photocycle (Fig. 3B) [20].

### Proton pumping activity tests and the expression of GR *in vivo*

**Figure 4:**
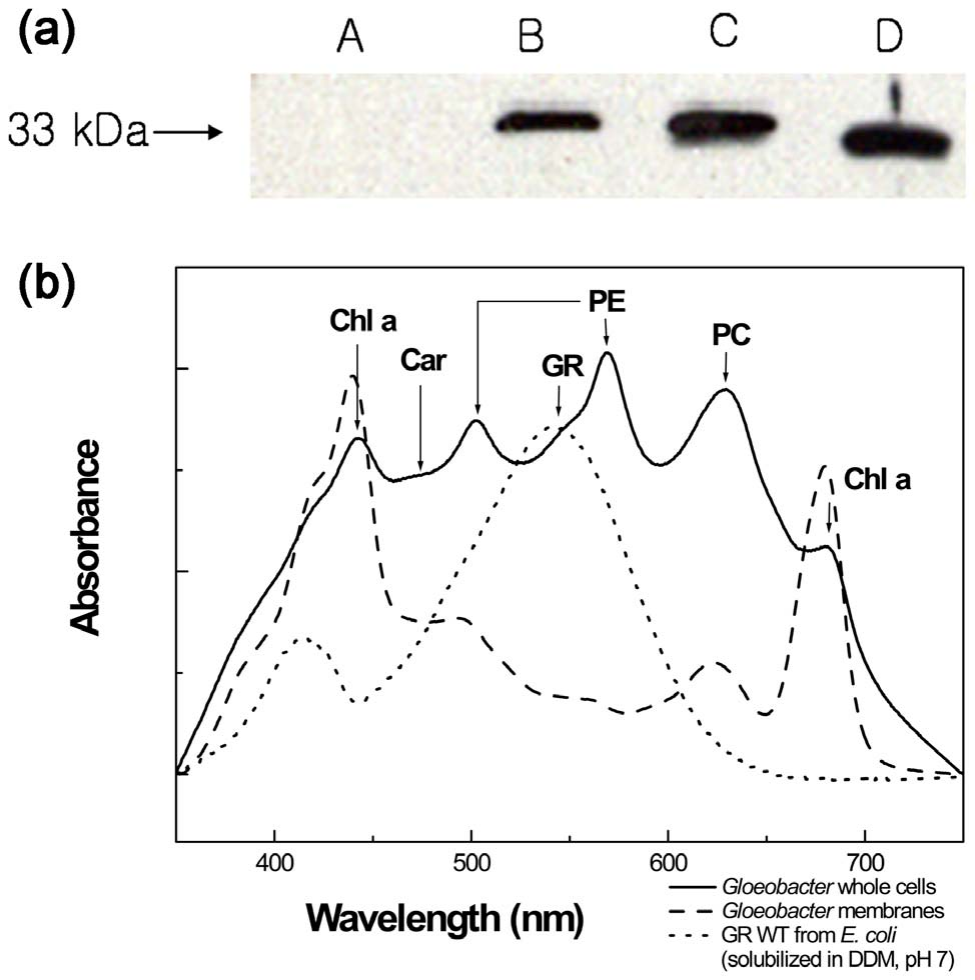
Immunoblot and spectroscopy analysis. (a) Immunoblot analysis of the *Gloeobacter* cells and the His-tagged GR protein. The immunoblot used anti-GR antibody for the membrane protein fraction of *Gloeobacter* cells and anti-His-tag antibody for the protein expressed in *E. coli*. The whole cells (C) were sonicated and lysates were ultracentrifuged. The lysates were separated into supernatant (A: soluble protein) and pellet (B: membrane protein). D: purified GR from *E. coli*. (b) Absorption spectra of intact cells and membranes of *G. violaceus*. The spectrum of *G. violaceus* is shown with solid line. Chl a (chlorophyll a), Car (carotenoid), PE (phycoerythrin), GR, and PC (phycocyanin) are marked by the arrows. The spectrum of *G*. *violaceus* membrane is shown by the dashed line. The spectrum of purified GR in 0.02% DDM, isolated from *E. coli* is shown by dotted line.

To verify whether GR is really expressed in the *Gloeobacter violaceus* cells we raised the antibody for GR in mice. Using western blotting, the band of 33 kDa was detected in the *Gloeobacter* whole cell membrane protein fraction (Fig. 4A), which correlated with the band of the recombinant protein expressed in *E. coli*. Thus, GR must be expressed in its native host at high levels. But the key question is whether GR has a function of proton translocation in *Gloeobacter* cell. Before testing the proton pumping activity we measured the visible absorption spectra of the *Gloeobacter* whole cells (Fig. 4B). Several peaks were observed and the 678 and 440 nm bands were assigned to Chl a. Phycoerythrin (PE) may show two peaks at 500 and 565 nm, which are similar to those for PE from red algae. Peak for phycocyanin (PC) was located at 620 nm. One band for carotenoid(s) was located at 480 nm, which was also apparent in cytoplasmic membranes [42]. Gloeorhodopsin (GR) showed peak at550 nm, clearly visible in the membrane fraction and observed as a shoulder in the cells spectrum. It is somewhat higher than the 540 nm maximum of the spectra of GR purified from *E. coli*, possibly because spectra of solubilized rhodopsins are usually blue-shifted relative to those of the membrane-embedded forms.

In *G. violaceus*, GR exhibits outward-directed light-induced proton transport. At pH 7, intact cells with GR carry out light-driven proton ejection. Illumination of *Gloeobacter* cells produces acidification of the medium, which is abolished by the protonophore carbonyl cyanide *m*-chloropheny-hydrazone (CCCP). Thus, the cells contain an outward-directed light-driven proton pump.

**Figure 5:**
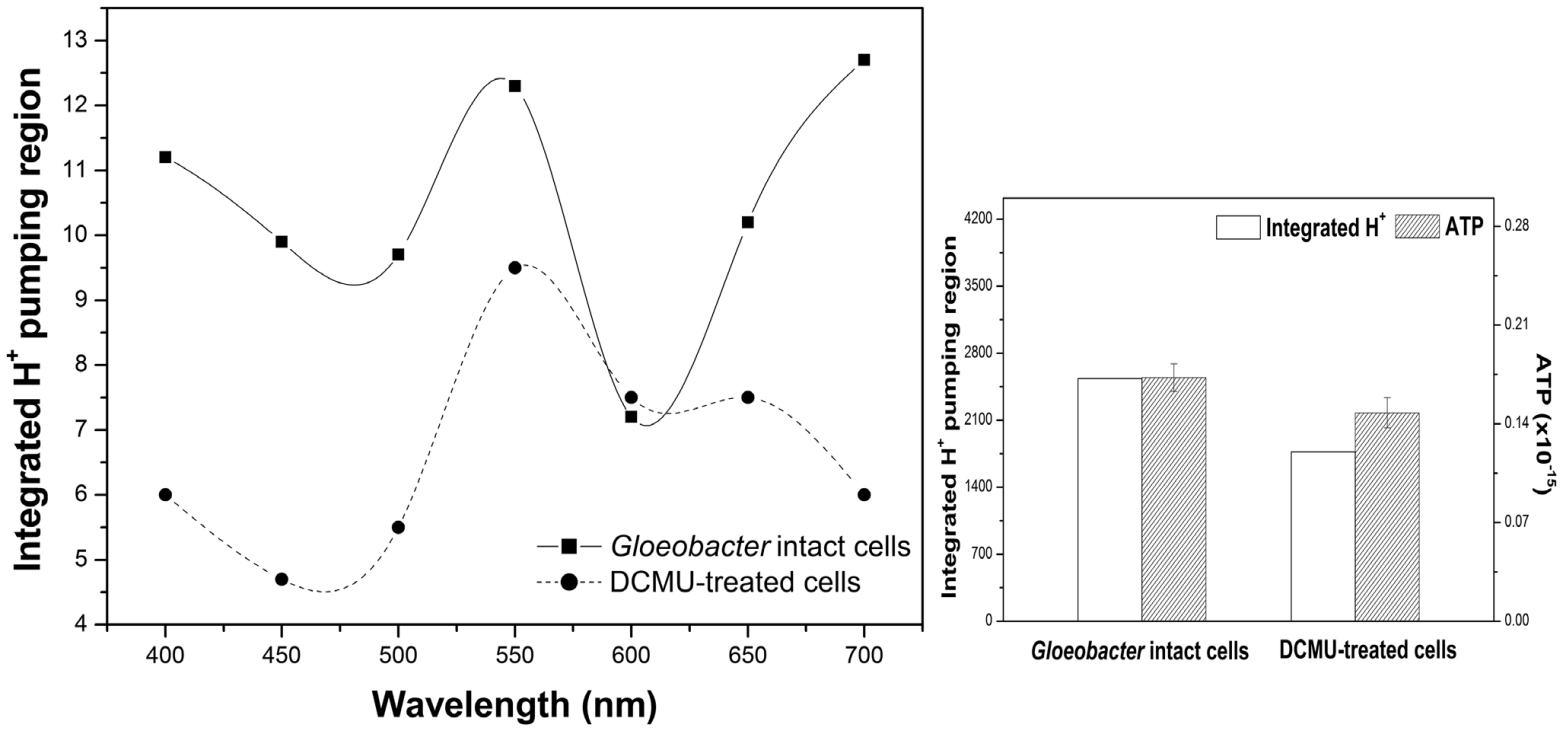
Action spectra for light-driven proton translocation in *Gloeobacter* cells (solid line). Sphaeroplasts of *Gloeobacter* cells were illuminated through the filters transmitting at 700, 650, 600, 550, 500, 450, and 400 nm. The efficiency of the sphaeroplasts (after the photosynthesis inhibition by DCMU treatment (5×10^−5^mol l^−1^)) was most efficient at 550 nm (dashed line). Integrated H^+^ pumping amount is presented by blank column. Concentration of synthesized ATP is presented by slashed column.

We treated the cells by well-known photosystem inhibitors, such as BQ, which accepts electrons from a site in the photosynthetic electron-transport chain after the photosystem II [43], and DBMIB, a quinine analogue. The reagents totally abolished the proton pumping activity of *Gloeobacter*, possibly due to general proton gradient dissipation. To obtain the action spectrum of the proton transport, *Gloeobacter* cells were illuminated through the filters (Sigma Koki SCF-400F40-10, 450F40-10, 500F40-10, 550F40-10, 600F40-10, 650F40-10, and 700F40-10). The proton pumping activity of *Gloeobacter* cells was wavelength-dependent, and most efficient at 400, 550, and 700 nm (Fig. 5).

After the treatment with DCMU (3-(3,4-dichlorophenyl)-1,1-dimethylurea, 5×10^−5^mol l^−1^), which is a specific inhibitor for photosystem II, the action spectrum of the proton transport showed that the proton pumping activity of sphaeroplasts of *Gloeobacter* cell was most efficient at 550 nm (Fig. 5). This may suggest that the proton transport is a result of GR's function. In addition, we tested ATP synthesis activity in the absence and presence of DCMU (3-(3,4-dichlorophenyl)-1,1-dimethylurea, 5×10^−5^mol l^−1^) using 5 min light illumination (>450 nm). It showed positive correlation between proton pumping activity and ATP synthesis in the cell (Fig. 5).

We also grew *Gloeobacter* cells in the presence of 1 mM nicotine, which is a retinal synthesis inhibitor [44]. The cells did not show the absorption peak at ∼540 nm, and eventually were dead under the light. Based on these results we may suggest that GR has a critical role in energy production of *Gloeobacter*.

## Discussion

We isolated gloeorhodopsin gene from *G. violaceus* PCC7421. The *Gloeobacter* rhodopsin gene encodes a polypeptide with a molecular weight of 33 kDa (Fig. 4A). GR and three mutants were expressed in *E. coli* and the pigments with 6-histidine tag were purified. The residues at position 121 (putative acceptor) and 132 (putative donor) were replaced, and their absorption spectra were compared. Absorption maximum of the WT GR pigment was about 540 nm at pH 7 and λ_max_ of D121N and E132Q mutant pigments were significantly red-shifted. This occurs because the residue at position 121 is close to the Schiff base nitrogen and the residue at position 132 is strongly coupled with the Schiff base [20], [21]. The mutations may lead to the changes of the local structure, producing a lower energy gap between the ground and excited state of the retinal.

Based on the sequence alignment, the retinal-binding lysine in helix G is Lys257 in GR, and the Schiff base counterion of BR Asp85 in helix C is Asp121 in GR. The pK_a_ of the counterion of the WT GR is about 5.9. In the D121N mutant, the pK_a_ of another group is 6.0, with very small spectral changes. Probably, another residue close to retinal (or coupled to it, as in the case of E132) is titrated with pK_a_ of 6.0. Interestingly, the position of the absorption maxima as a various pH of E132Q (BR's D96) is significantly similar to one of BR WT. It has been observed earlier that upon lowering the pH, BR undergoes characteristic changes. BR has the acid blue form (BR_AB_) [45], [46] and the acid purple state (BR_AP_) in lower pH and presence of Cl^−^ or other halide ions [47]. Chloride ion binds to Bacteriorhodopsin at low pH [48]. A result of measuring the spectra of GR E132Q at pH 3 in 1 M NaCl was red-shifted (6 nm) compare to GR E132Q in the absence of Cl^−^. In GR E132Q, an anion might show a similar effect like BR.

Proton pumping activity of the WT GR was observed under the light. On the other hand, the proton pumping activity of the D121N and E132Q is poor. The former, D121N can be explained by the blockage completely. Proton uptake and release are not totally abolished for E132Q like for D121N. The saw tooth pattern may be explained by severe retardation of the intra-membrane proton translocation.

GR shows a rapid photocycle and produces K, M, and O intermediate. We observed transient flash-induced absorption changes attributable to a photochemical reaction cycle which is characteristic of retinylidene ion pumps and similar to the photocycle seen with *E. coli* membranes expressing proteorhodopsin (a photochemical reaction cycle with characteristic time of 47 ms) [26]. The GR protein is produced in *Gloeobacter* cells, as the pigment is readily detected in *Gloeobacter* by immunoblot analysis (Fig. 4A), even though *Gloeobacter* is a photoautotroph. Proton pumping activity of the *Gloeobacter* cells was observed maximally upon excitation at around 550 nm and 700 nm, most probably from GR and the photosynthesis system, respectively. We suggest that GR pumps protons out of the cell together with the chlorophyll-based machinery.

After the DCMU treatment, the proton pumping activity of *Gloeobacter* cells was most efficient at 550 nm, suggesting that it comes from GR, even though some contribution from PE-mediated proton transport by PS-I coupled to cyclic electron transport is possible.

As microbial rhodopsins were discovered in various organisms, it is possible that a rhodopsin precursor existed before the kingdoms diverged. Although *Gloeobacter* is a cyanobacterium, it lacks thylakoid membrane development [18], and its photosynthetic electron transfer system co-exists in the cytoplasmic membrane with rhodopsins. As the efficiency of chemiosmotic coupling should be low in the absence of thylakoids, one may need a rhodopsin to help produce proton gradient in the periplasm. In *Gloeobacter*, GR absorbs at 540 nm, and photosystems absorb at red wavelength regions, where the pumping of H^+^ ions and the ensuing conversion of ADP + Pi into ATP is driven by electron gradients established in the membrane. So, GR could compensate for the poor energy production from photosynthetic machinery (Fig. 5). Another example of a similar *Roseiflexus* rhodopsin in bacteria without chlorosomes could support the idea about co-existence of opsins with photosynthetic machinery. In addition, GR showed a carotenoid binding activity like XR [30]. One of the carotenoids in *Gloeobacter* cell, which is echinenone, could bind GR [49]. It is also supported the idea of energy conversion of various wavelength of light. *Gloeobacter* used to convert light energy to proton motive force by photosynthetic machinery with phycobillin plus rhodopsin with carotenoid antenna. Recently Vogt et al. [50] reported *Gloeobacter* rhodopsin, limitation of proton pumping at high electrochemical load, which is explained the function of GR could be to sustain a provisional membrane voltage under conditions where chlorophyll-based photosynthesis is depleted. We may suggest that *Gloeobacter* fills the evolutionary gap between rhodopsin-based energy production and photosynthesis. On the other hand, we also could not exclude the possibility that the coexistence of retinal and chlorophylls is a result of a relatively recent lateral gene transfer, similar to several other rhodopsins.

**Figure 6:**
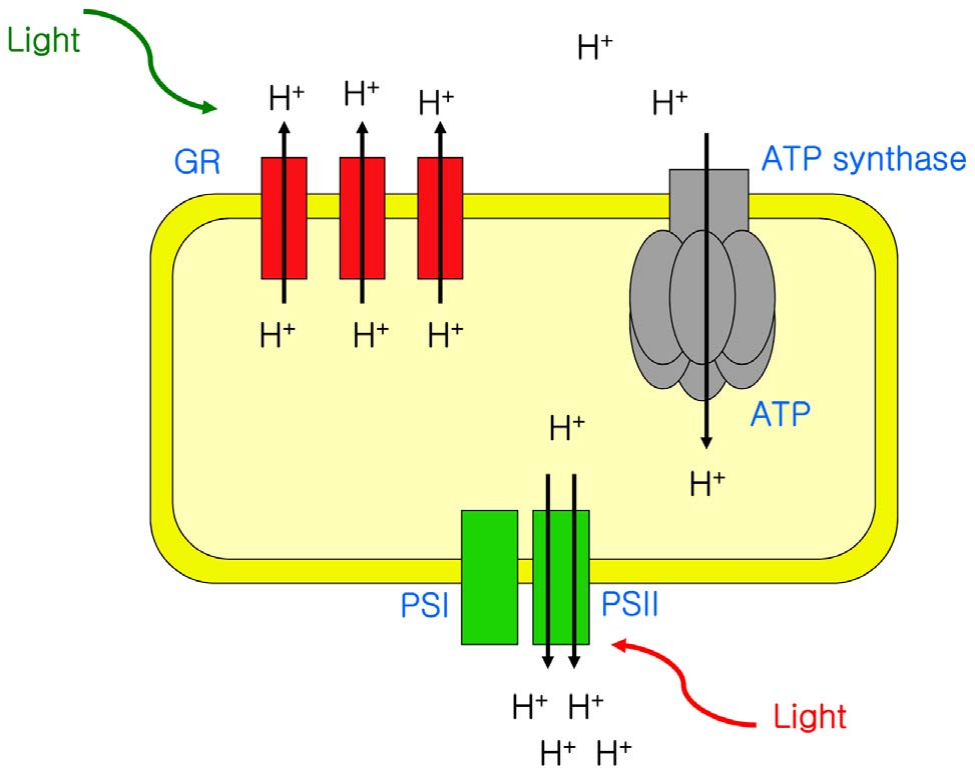
Schematic model of energy production in *Gloeobacter*. The photosynthetic apparatus of *Gloeobacter* is reproduced from [51].

To sum up, *Gloeobacter* has two types of light-driven proton pumps – chlorophyll-based photosystems and rhodopsin. A schematic model of the energy production with both rhodopsin and photosynthesis is shown in Fig. 6.
